# Purification of vegetable oils from acrylamide pollutants using metal-organic frameworks

**DOI:** 10.1038/s41598-025-28117-z

**Published:** 2025-11-29

**Authors:** Reda M. Abdelhameed, Randa S. Hasan, Hassan Abdel-Gawad

**Affiliations:** 1 Applied Organic Chemistry Department, Chemical Industries Research Institute, National Research Centre, 33 EL Buhouth St., Dokki, Giza, 12622 Egypt; 2 Regional Centre for Food and Feed (RCFF), Agricultural Research Center (ARC), P. Box 588, Orman, Giza, Egypt

**Keywords:** Metal-organic framework, Acrylamide, Sunflower oil, Acid value, Peroxide value, Chemistry, Engineering, Environmental sciences, Materials science

## Abstract

Acrylamide is a chemical compound that can form in certain foods during high-temperature cooking processes like frying, roasting, and baking. The presence of acrylamide in used cooking oil has environmental impact; therefore, the purification of used oils may reduce the environmental risks. Nowadays, biochar, a porous carbonaceous material derived from biomass pyrolysis can added to crystalline porous materials called metal-organic framework (MOF), these combination lead to fantastic properties for adsorption of contaminants. Here, ZIF-8@Biocharas adsorbents were synthesized for purification of the used frying oil from many pollutants, including, acrylamides, acids and peroxides. First, surface and pore properties, and morphologies of the adsorbents were determined using different characterization techniques. The used frying oil was treated with 0.5% (w/w) ZIF-8@Biochar and ZIF-8 MOF to remove free fatty acid (FFA), the results showed reduction by 80.6% and 32.2%, respectively. Likewise, peroxide value reductions ranged from 70.6% with the same adsorbent. The residues of acrylamide were determined by GC-MS/MS. whereas acid and peroxide values were determined by titration methods. This study showed an economical solution for reducing acrylamide, acid value, and peroxide value in used cooking oil for improving safety and quality of used oil.

## Introduction

Waste cooking oil has a harmful effect on environment and human health due to the formation of acrylamide^1^. The inappropriate disposal methods for waste cooking oil cause environmental pollution^2^. Acrylamide formation in food by Maillard interaction, which interacts with asparagine (amino acid) and glucose (reducing sugars) under a high temperature rise of 120 °C, especially food containing starch^3^. Studies measuring acrylamide in specific fried oils have found levels ranging from approximately 779 µg/kg to 1299 µg/kg, depending on the type of oil and frying conditions. For example, a study on different oils found the following ranges after multiple frying sessions: sunflower oil (890–1200 µg/kg), olive oil (892–1163 µg/kg), corn oil (981–1299 µg/kg), and hazelnut oil (779–1120 µg/kg)^4,5^. Aladedunye ‎et al.^6^ revealed that acrylamide found in frying oil because of oils interaction with carbonyl groups and amino acids form food which frying through high temperature.

Acrylamide has neurotoxic effect on humans, reproductive toxicity, immunotoxicity, stimulates apoptosis in mitochondria, and hepatotoxicity^7,8^. Acrylamide was classified as probable human carcinogen by International Agency for Research on Cancer (IARC)^7^. Waste cooking oils contain a high concentration of acrylamide and reused this oils caused cardiovascular disease and liver disease for human^9,10^. There are many methods used for purifying cooking oil, such as phosphoric acid treatment, hot distilled water and neutralization with alkali, but it is difficult to remove acrylamide from oil because it is a complex chemical that forms at high temperatures, not a simple impurity like free fatty acids or pigments. Instead, reducing acrylamide requires preventive measures or specific post-treatment methods like using certain adsorbents, as it is a result of high-temperature reactions in the food being cooked, not a standard impurity in the raw oil itself^2,11^.

Adsorbents such as activated carbon and biomaterials can remove acrylamide from oils^12–16^. The agricultural by-product, sugarcane residue, has been shown to be effective in removing contaminants from used cooking oils, including acrylamide. Cellulose from the water hyacinth plant has been used as an adsorbent for acrylamide in frying oil. Adsorbents such as starch-grafted polymers have shown high affinity for contaminant removal and can be economical and biodegradable. Other biomaterials, such as those derived from corn straw, have been explored for their ability to remove acrylamide, particularly in aquatic environments, which may inspire similar applications for oils. The reported mechanisms for removing acrylamide molecules using adsorption methods are governed by: (i) physical adsorption, where the sorbent has a porous structure with a large surface area that traps acrylamide molecules; (ii) surface interactions, where the chemical properties of the sorbent surface, such as the presence of hydroxyl, carboxyl, or amide groups, can attract and bind acrylamide molecules through various interactions.

Metal-organic frameworks (MOFs) are a class of crystalline porous materials composed of metal ion clusters linked together by organic molecules^17–24^. MOFs can be synthesized using various methods, including microwave-assisted synthesis, colloidal deposition, and solvothermal/hydrothermal methods^17–29^. The unique structure features such as high porosity and very large internal surface area, allowing MOFs to be applied in a wide range of applications^30–32^, recently, MOFs are promising materials for vegetable oil purification. MOFs such as aluminum and titanium, are used for purifying unrefined vegetable oils and getting better taste and odor because of binding peroxide and free fatty acids^33^. MOFs can act as adsorbents, selectively binding to and removing contaminants like free fatty acids, peroxides, and color pigments. MIL-101 MOF has been used to adsorb herbicides from edible oils, and MIL-53(Al), Zn-MOF, and MIL-125(Ti) has been used to refine crude vegetable oil. MOFs can be more effective and easily regenerated than traditional adsorbents. Further research is needed to optimize MOF performance and stability for specific oil purification applications.

A composite material combining MOFs, specifically ZIF-8, with biochar offers a novel and effective solution to address the individual shortcomings of each component. By integrating these two materials, we can create a synergistic composite with enhanced adsorption properties. In the present work, nanometric ZIF-8 was prepared and incorporated in biochar to facilitate the purification process of used cooking oils by removing impurities such as acrylamide, free fatty acids (which cause high acidity), and peroxides. This process leverages the synergistic properties of both materials: the high surface area and tunable pores of ZIF-8 combined with the cost-effectiveness and porosity of biochar.

## Materials and methods

### Materials

The two types of used cooking oils (sunflower oil and mixed soybean and sunflower oil) were collected from local market in Cairo, Egypt. Acrylamide was purchased from Sigma Aldrich. 2-Methylimidazole (Hmim), Zinc nitrate [Zn(NO_3_)_2_], methyl alcohol, ethyl alcohol, hydrochloric acid (HCl), sodium hydroxide (NaOH), and all other chemicals were purchased from Sigma as analytical-grade reagents.

### Preparation of biochar

Date was purchased from local markets in Cairo, Egypt. The date pits were collected after physical separation of the date flesh fruit. Date pits (100 g) were washed with 1 L distilled water, then 500 mL ethanol several times to remove impurities. The date pits were dried at room temperature for 2 days then dried at 50 °C for 24 h in an oven. The dried date pits were heated in a furnace at 550 °C for 3 h. The resultant biochar was crushed and sieved. Biochar samples were washed with deionized water to remove fine particles and soluble salts. Then, date pits were washed with 50 mL of 37% concentrated hydrochloric acid for 2 h. The biochar samples were oven-dried at 105 °C for 2 h and stored in airtight containers.

### Synthesis of zeoliticimidazolate frameworks-8 (ZIF-8)

ZIF-8 was synthesized according to Shi et al.^34^ with slightly modification as follows: 2-methylimidazole (6.56 g, 0.08 mol) was dissolved in methanol (120 mL) then added to Zn (NO_3_)_2_·6H_2_O (2.95 g, 0.01 mol) dissolved in in 60 mL of methanol. The reaction mixture was stirred for 20 min, filtered off, washed with methanol several times. The resulting white precipitate was dried in an oven at 80 °C until used.

### Synthesis of ZIF-8@Biochar

**Fig. 1:**
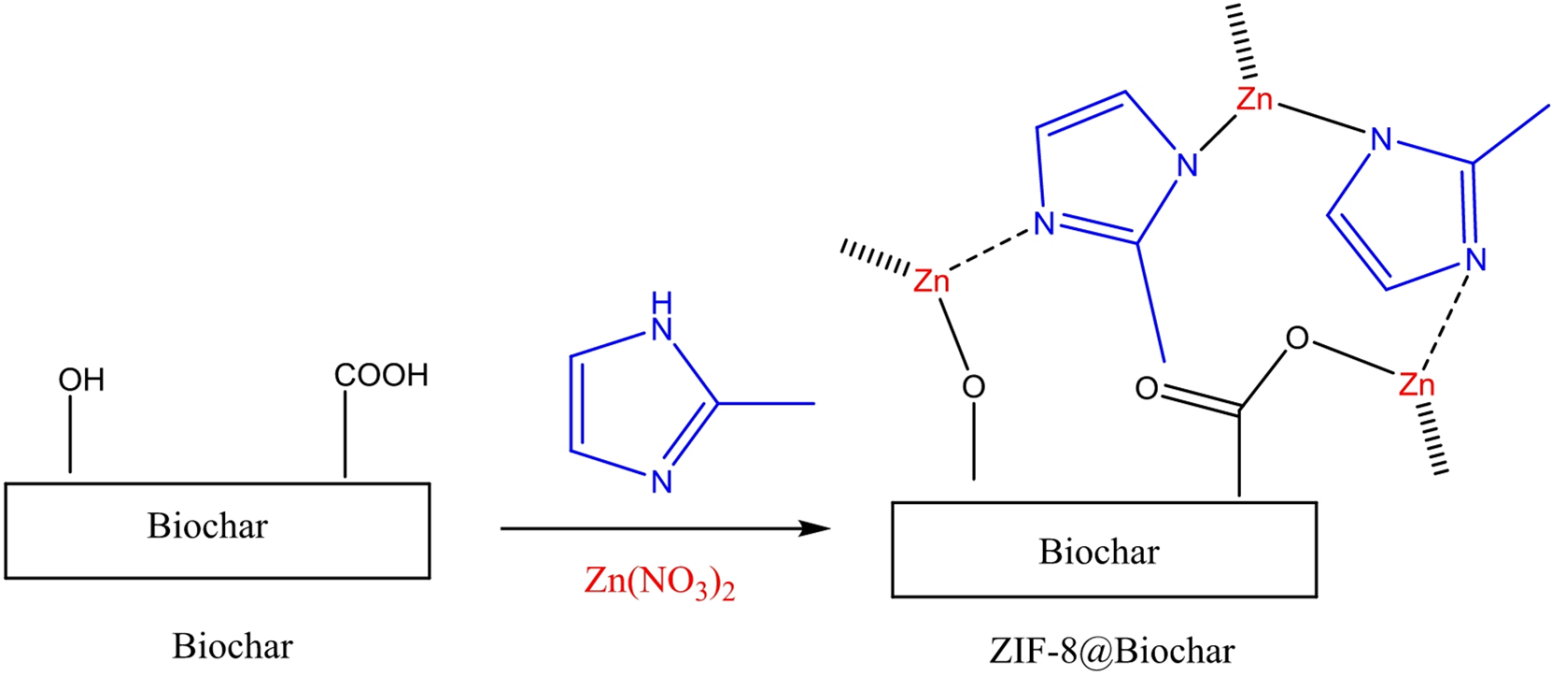
Synthesis of ZIF-8@Biochar.

ZIF-8@Biochar composites were created as follows: 2-methylimidazole (6.56 g, 0.08 mol) in methanol (120 mL) was added to Zn (NO_3_)_2_·6H_2_O (2.95 g, 0.01 mol) in 60 mL of methanol, followed by addition of 4.00 g of biochar. The reaction mixture was stirred overnight at room temperature, filtered off, washed with methanol several times. The resulting precipitate was dried in an oven at 80 °C until used (Fig. 1).

### Characterization of materials

High resolution scanning electron microscopy was used to examine the morphological structures ofZIF-8 alone, biochar, and ZIF-8@Biochar (HRSEM Quanta FEG 250 with field emission gun, FEI Company - Netherlands). Energy dispersive X-ray spectroscopy (EDX) analysis unit (EDAX AME-TEK analyzer) coupled to the same microscope was used to record the elemental analysis. Cu K X-radiation at 40 kV, 50 mA, and = 1.5406) at room temperature, where diffraction data were acquired in steps of 2° ranging from 4° to 50° with a step size of 0.02° and scanning rate of 1 s, allowed X-ray diffraction (XRD) to be observed for the samples. The materials were also examined using a Japanese JASCO FT/IR-4700 spectrophotometer to perform Fourier Transform Infrared Spectroscopy (FTIR) analysis. The FTIR measurement parameters were 4000 –400 cm^− 1^ wave number range, the number of scans was 16 and resolution were 4 cm^− 1^. The sample preparation method was adding 2 mg solid sample to 10 mg KBr and pressed as pellet for measurements. The physicochemical attributes of biochar/MOF nanocomposites, encompassing pore size, surface area, and grain structure, were evaluated through Brunauer-Emmett-Teller (BET) analysis, which was performed using a Micromeritics^®^ ASAP™ 2020 instrument, coupled with TriStar™ II version 2.00 software, both supplied by Micromeritics Instrument Corporation.

### Treatment of oils with MOFs

Two types of used oil (sunflower and mixed oil from soybean and sunflower) were used for purification process. All experiments were performed three times at 25 °C in an air atmosphere; the oils (10 mL) was mixed with 20 mL hexane and the MOF (50 mg) as an adsorbent in a glass flask under stirring for different times intervals (1, 2, 3, 4,and 5 h). The liquid phase was then separated from the adsorbent by filtration and analysis.

### Determination of acrylamide in oils by GC/MS/MS

#### Extraction

Acrylamide was extracted from the used oil according to the described protocol^35^. The used oil samples (25 mL) were mixed with 75 mL of water into beaker glass. The mixture was mixed and put in water bath 70 °C for 30 min then 40 mL of 1-propanol was mixed then the mixture was centrifuged. The supernatant (after centrifugation) was put into mark flask then evaporated by a rotary ‎evaporator under 50 Torr. Acetonitrile (3 mL) and hexane (20 mL) were added to sample and mixed by an ultrasonic bath. The acetonitrile layer was put in reagent glass closed by screw cap until analysis.

#### Sample analysis through GC/MS/MS

The GC-MS-MS instrument was connected with a mass selective detector used to determine acrylamide (Agilent Technologies 7890 A- MSD Agilent 7000), which contend 5%-phenyl methyl poly siloxane (HP-5ms Agilent) capillary ‎column (30 m × 0.25 mm i.d and film thickness 0.25 μm). Helium was used as carrier gas with a linear velocity 1 mL per min. The temperature of oven was started 100 °C for two min, then 285 °C at a rate of 50 °C per min. The temperatures of the injector are 250 °C, and the detector was 250 °C. The split: split ratio 1: 20 was injection mode, with a volume of one µL. The MS operating parameters were interface temperature 280 °C and ionization potential 70 eV. The mode of selected ion monitoring m/z (SIM) 44, 55, and 71 was used^35^.

#### GC-MS-MS validation

The recovery was calculated by comparing the measured concentration of spiked samples with the known added concentration acceptable recovery values ranged between 80 and 110%, depending on the matrix and analyte. The mean recovery of acrylamide from spiked sunflower oil and mixture oil sample was found to be (96.3–100.6%) respectively, which is within the acceptable number.

$${\mathbf{Recovery}}{\text{ }}\left( \% \right)={\text{ }}\left( {{\text{Measured }}-{\text{ Original}}} \right){\text{ }}/{\text{ spiked }} \times {\text{ 1}}00$$

**Accuracy** was assessed by analyzing spiked samples at two concentration levels, and comparing the measured values to the nominal values. The bias was showed acceptable accuracy, with bias values ranging from 4.3 to 10.6%, Accuracy % (95.7–89.4%).

$${\text{Bias }}\left( \% \right){\text{ }}={\text{ }}\left( {{\text{Measure }}-{\text{ True}}} \right)/{\text{ True }} \times {\text{ 1}}00$$

$${\mathbf{Accuracy}}{\text{ }}\left( \% \right)\,=\,{\text{1}}00\% {\text{ }} - {\text{ Bias }}\left( \% \right)$$

**Precision** was expressed as relative stander deviation (RSD %). The method showed precision with RSD values l between (2.81–3.39%), which than 5% across all tested concentration levels. The method was validated according FAD (2018)^36^. **The linearity** was assessed over the concentration range of three concentrations. The method showed linearity, with a correlation coefficient (R^2^) of 0.9989, which is within the general accepted limit (≥ 0.99). **The limit of detection** (LOD) and limit of quantification (LOQ were determined based on the stander deviation of the response (SD) and slope of calibration curve using the following equation:

$$LOD=\frac{{3.3~ \times ~SD}}{{Slope}}\,\,\,LOQ=\frac{{10~ \times ~SD}}{{Slope}}$$

The limit of detection (LOD) and limit of quantification (LOQ) were calculated (3.59 and 10.98 µg/ kg), respectively.

### Determination of acid values in used cooking oils

The acid value of used cooking oil is determined by titrating a solution of the oil with a standardized alcoholic solution of potassium hydroxide (KOH)^37^. The procedure was as follow: sample preparation, 10 mL of the used cooking oil sample was dissolve it in 10 mL ethanol until complete dissolution. Titration, add 1 mg of phenolphthalein indicator to the solution. Slowly add the standardized KOH solution (0.02 N) from a burette. End point detection; continue adding the base until a permanent, stable color change is observed, indicating that all free fatty acids have been neutralized. This is the end point of the titration. Calculation, use the volume of the base used to calculate the acid value. The formula is:

$${\text{Acid Value }}={\text{ }}\left( {{\text{V}} \times {\text{N}} \times {\text{MW}}} \right) \times {\text{1}}000$$

*V* = volume of KOH solution used (in mL), *N* = normality of the KOH solution, *M* = molecular weight of KOH (56.1 g/mol), *W* = weight of the oil sample in grams.

### Determination of peroxide values in used cooking oils

The peroxide value of used cooking oil was determined by measuring the amount of peroxides, which indicates rancidity and lipid oxidation, using iodometric titration^37^. The oil sample (0.3 mL) was dissolved in 10 mL a mixture of acetic acid and chloroform (1:1 v/v), then saturated Kl solution was added. The mixture was put for five minutes in the dark, and then put distilled water (20 mL) with shake. Na_2_S_2_O_3_ solutions (0.01 N) were used for titrated liberated iodine. 1 mg starch was used as a colorimetric indicator, which turns blue-black in the presence of iodine and becomes colorless when all the iodine is consumed.

$${\text{Peroxide value }}\left( {{\text{milliequivalents}}/{\text{kg}}} \right){\text{ }}=\frac{{\left( {Vs-Vb} \right) \times ~F~ \times ~10~~}}{W}$$

Where, *Vs* (titration volume of sample mL); *Vb* (titration volume of blank mL); *F* (factor of 0.01 N Na_2_S_2_O_3_ solution); *W* (weight of fat in volume of extract used g); N (normality of Na_2_S_2_O_3_ solution).

### Statistical analysis

Standard deviation (SD) and standard error (SE) were used for statistical analysis by (Fisher, 1970)^38^. Significant differences between results were compared by Least Significant Difference test (LSD) and Waller and Duncan, 1969^39^. All of the static analysis carry out by Costat program.

## Results and discussion

### Morphology and structure characterization of the adsorbent composite

**Fig. 2:**
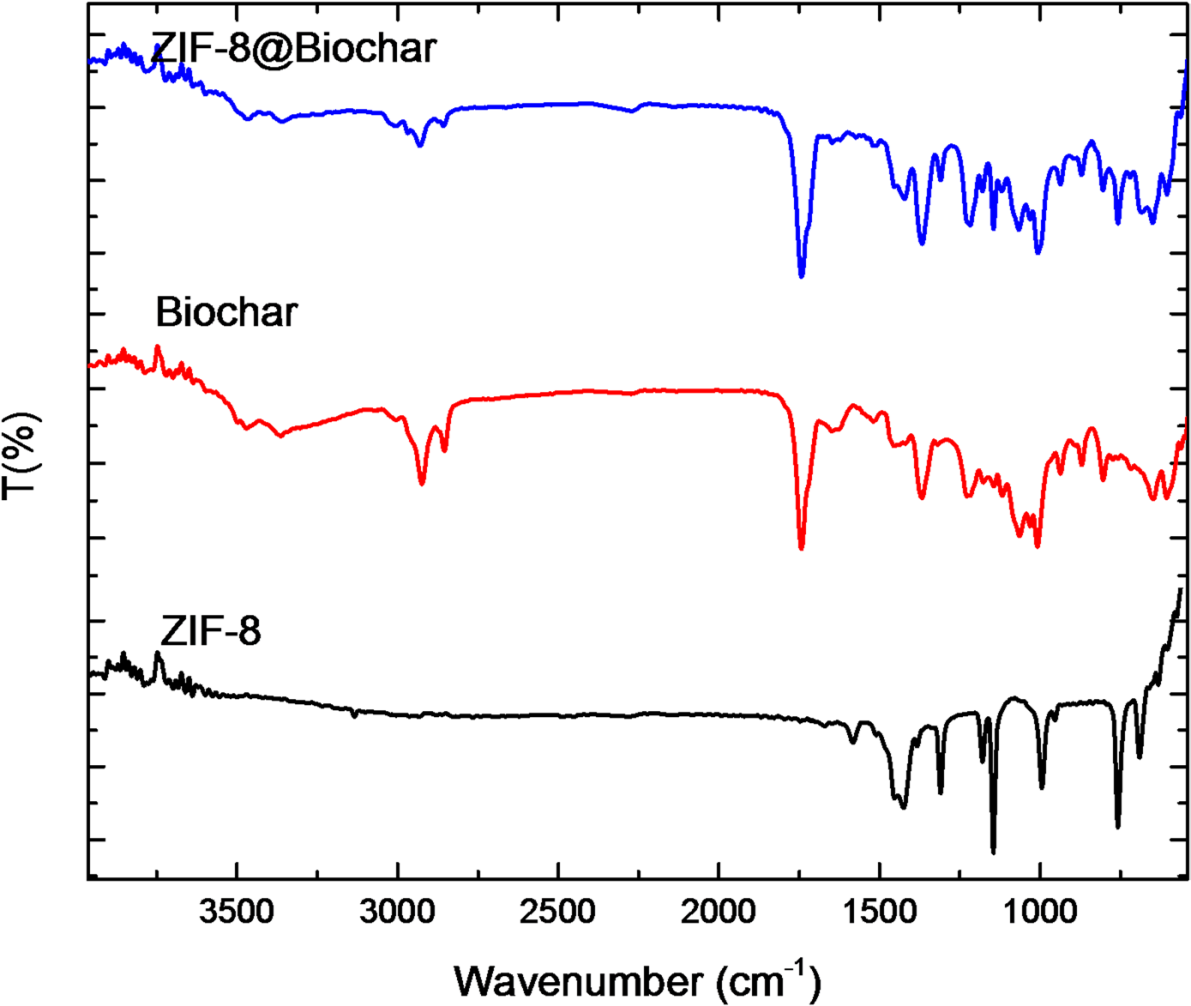
FTIR of ZIF-8 alone, biochar, and ZIF-8@Biochar.

To describe the probable interactions in the composite, FT-IR was used. Figure 2 displays the ZIF-8 alone, biochar, and ZIF-8@Biochar. A FTIR spectrum of ZIF-8 exhibits several key absorption bands associated with the vibrations of its structural components: Zn-N stretching (around 421–427 cm^− 1^), imidazole ring vibrations (around 950–1350 cm^− 1^), C = N stretching (around 1584 − 1635 cm^− 1^), C-H stretching aromatic (around 3134–3135 cm^− 1^), C-H stretching aliphatic C-H (around 2927–2929 cm^− 1^). A FTIR spectrum of the biochar showed the absorption band at around 3430 cm^− 1^ connected to -OH groups and absorption band at around 2927 cm^− 1^ related to the C–H stretching of the aliphatic chain. Furthermore, biochar molecules showed peaks at 1700–1660 cm^− 1^ associated with carbonyl bond vibrations in the carboxylic group.

FTIR spectrum of a ZIF-8@Biochar composite showed a combination of the characteristic peaks for both ZIF-8 and biochar. A strong, sharp peak at approximately 421–427 cm^− 1^ confirms the zinc-nitrogen (Zn-N) bond from the ZIF-8 framework. Vibrations between 950 and 1350 cm^− 1^ correspond to the in-plane bending of the imidazole ring. Absorption bands around 3135 cm^− 1^ are from the aromatic C-H stretch, and peaks near 2929 cm^− 1^ come from the aliphatic C-H stretch of the methyl group in the 2-methylimidazole linker. A peak around 1584–1643 cm^− 1^ represents the stretching vibration of the C = N bond within the imidazole ring. Additionally, the spectrum for ZIF-8@Biochar contain peaks related to its carbonaceous structure and residual functional groups, such as carboxyl (-COOH) and hydroxyl (-OH) groups around 3100–3500 cm^− 1^ and 1540–1650 cm^− 1^ attributed to the O-H stretching of hydroxyl groups and C = O stretching in carboxylic acids, respectively. The good evidence of successful composite formation was some shifts or changes in intensity of some functional groups. The composite spectrum displayed enhanced peak at 1064 cm^− 1^ related to C-N, also the spectrum showed slightly shift compared to the pure starting materials. This indicates a molecular interaction or bonding between the ZIF-8 and biochar, confirming a true composite structure was formed, not just a physical mixture.

**Fig. 3:**
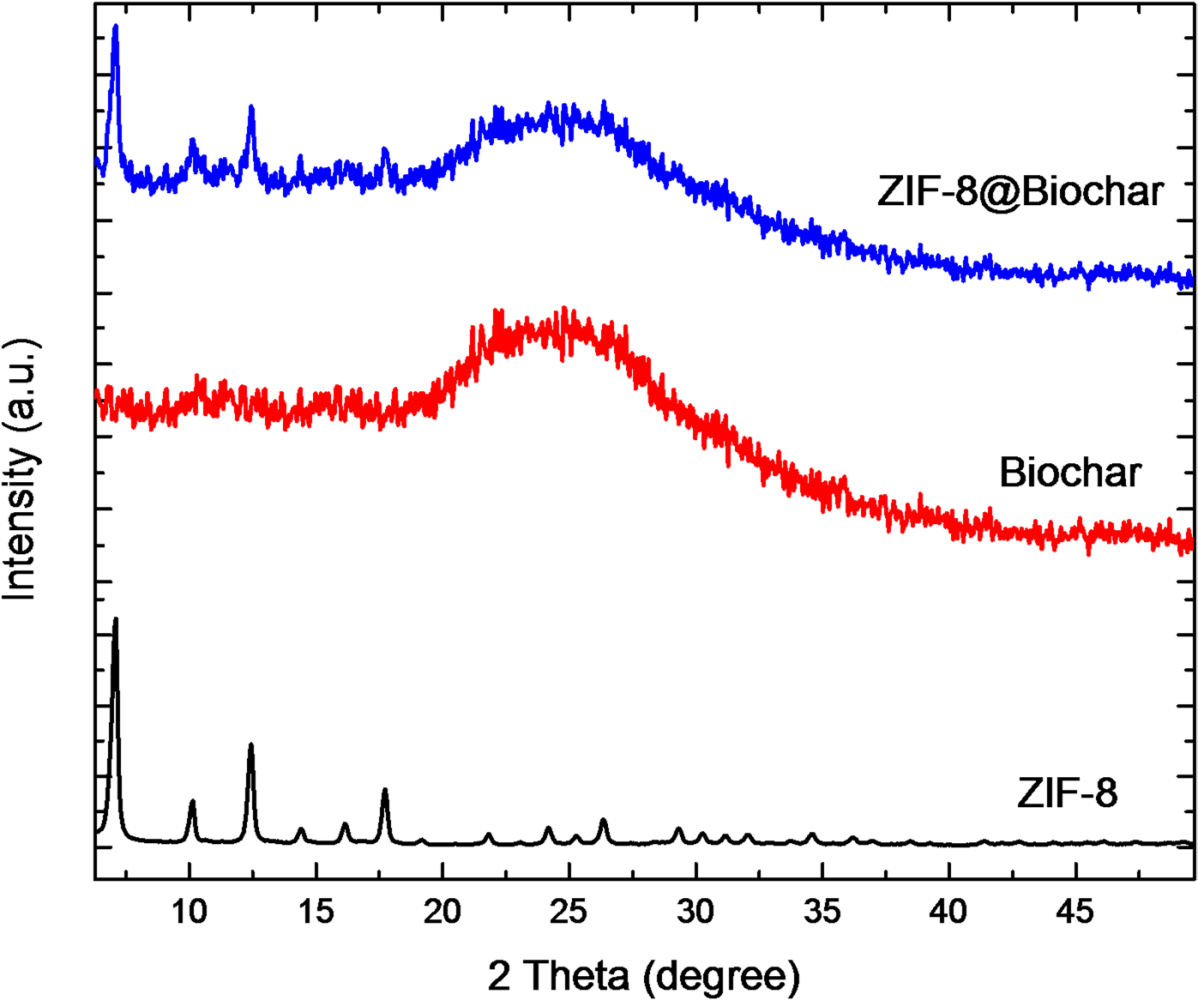
PXRD of ZIF-8 alone, biochar, and ZIF-8@Biochar.

Figure 3 illustrates the XRD patterns of the ZIF-8 alone, biochar, and ZIF-8@Biochar. The ZIF-8 XRD pattern matches previous reports^40,41^ indicating that ZIF-8 nanocrystals were successfully synthesized. The 2θ values for ZIF-8 showed peaks at 7.3°, 10.3°, 12.7°, 14.8°, 16.4° and 18.0°. The PXRD for biochar showed a broad peak in the low angle region (20º–30°), it was indexed as (002) related to stacking of the graphitic basal plan in biochar, confirming the successful preparation of biochar from date pits^42^. Both ZIF-8 and biochar have peaks in the XRD patterns of the ZIF-8@Biochar compounds. The crystallinity of ZIF-8@Biochar compounds is indicated by the sharpness and intensity of their XRD patterns, indicating that ZIF-8 nanocrystals were successfully grown in biochar solution during in situ synthesis and that biochar incorporation did not destroy the framework integrity of ZIF-8.

**Fig. 4:**
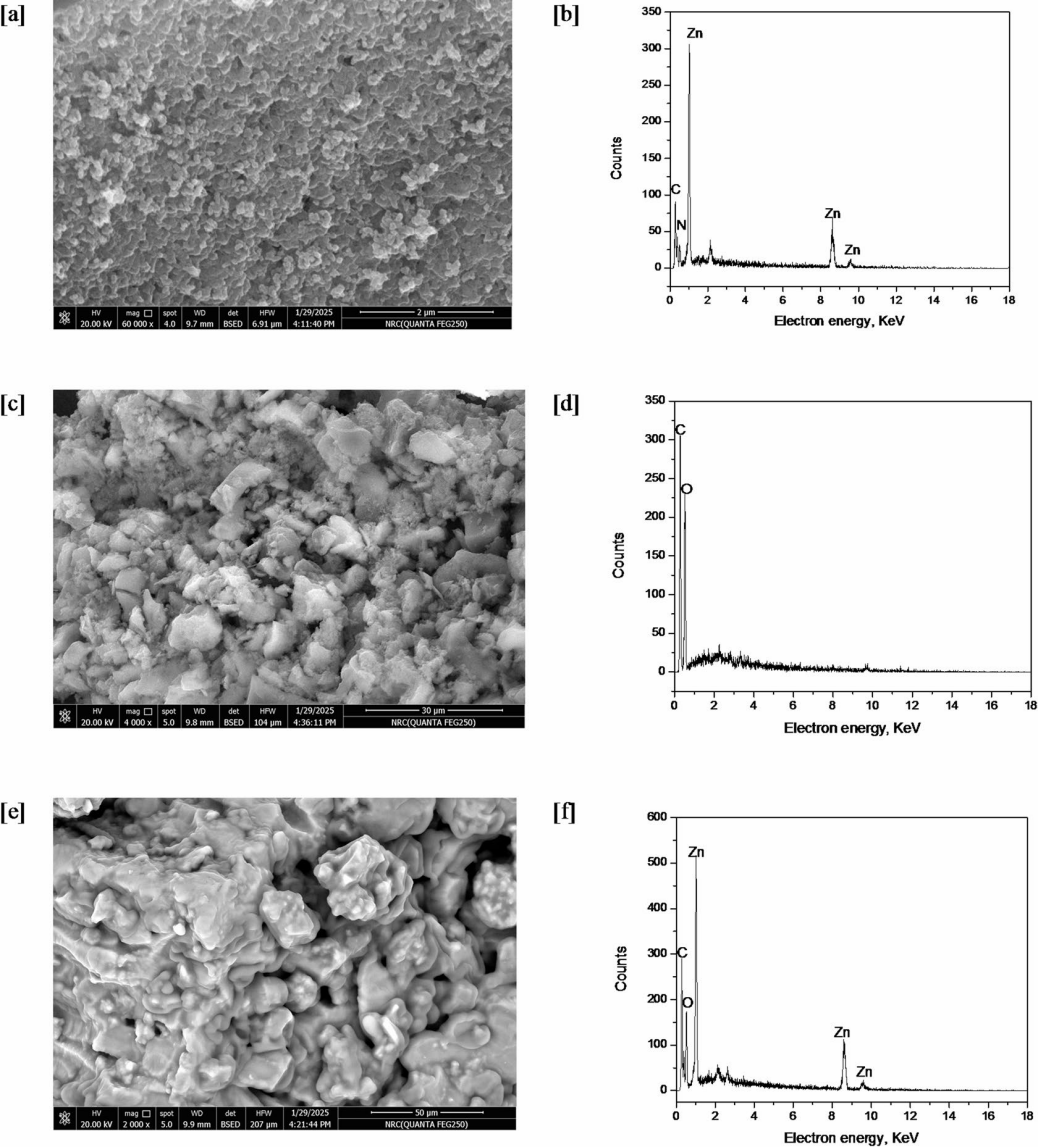
SEM and EDX of ZIF-8 alone [a, b], biochar [c, d], and ZIF-8@Biochar [e, f].

The study of the typical ZIF-8 loaded biochar composite using electron microscopy revealed morphological details and proved the existence of nanoparticles. Depending on the experimental conditions employed to generate them, the particles were shown to be spherical, distinct, and regular (Fig. 4). The particle sizes were around 100 nm. However, the composite depicts a surface that is smooth. It was possible to characterise the EDX. It enables measurement of the active molecules kept in the composite. Figure 4 displays the results of the EDX study of ZIF-8 alone, biochar, and ZIF-8@Biochar. EDX analysis of pure pristine ZIF-8 (Fig. 4b) confirms its elemental makeup, identifying the presence of zinc (Zn), carbon (C), and nitrogen (N). Biochar was primarily constituted of oxygen, carbon, and hydrogen, according to an EDX examination (Fig. 4d). The composite for ZIF-8@Biochar was fairly similar in terms of their chemical structure, with the exception of nitrogen and zinc, which are thought to be the most important elements in the composite (Fig. 4f). Due to the inclusion of ZIF-8 in the composite, these findings made sense.

Table 1 discloses the surface area, pore size, and grain size of the biochar, and ZIF-8@Biochar nanocomposites as determined by BET analysis. The surface area of the biochar increased from 105 m^2^g^− 1^ to 505.1 m^2^g^− 1^ when incorporated with ZIF-8, suggesting that the incorporated process amplified the surface area. Moreover, it indicates that the pores on the biochar surface might have been blocked by some ZIF-8 nanoparticles, leading to a fall in the pores size of the nanocomposite. The granular particle size was decreased when incorporated with ZIF-8 and increased their external surface area.

**Table 1:** Surface properties of biochar and ZIF-8@Biochar nanocomposites.

Adsorbent	Surface area S_BET_ (m^2^/g)	*BJH pore size (nm)	Grain size (nm)
Biochar	105.3	3.66	45.5
ZIF-8@Biochar	505.1	2.02	15.2

*BJH − Barrett-Joyner-Halenda approach.

### Removal of acrylamide from oil

**Fig. 5:**
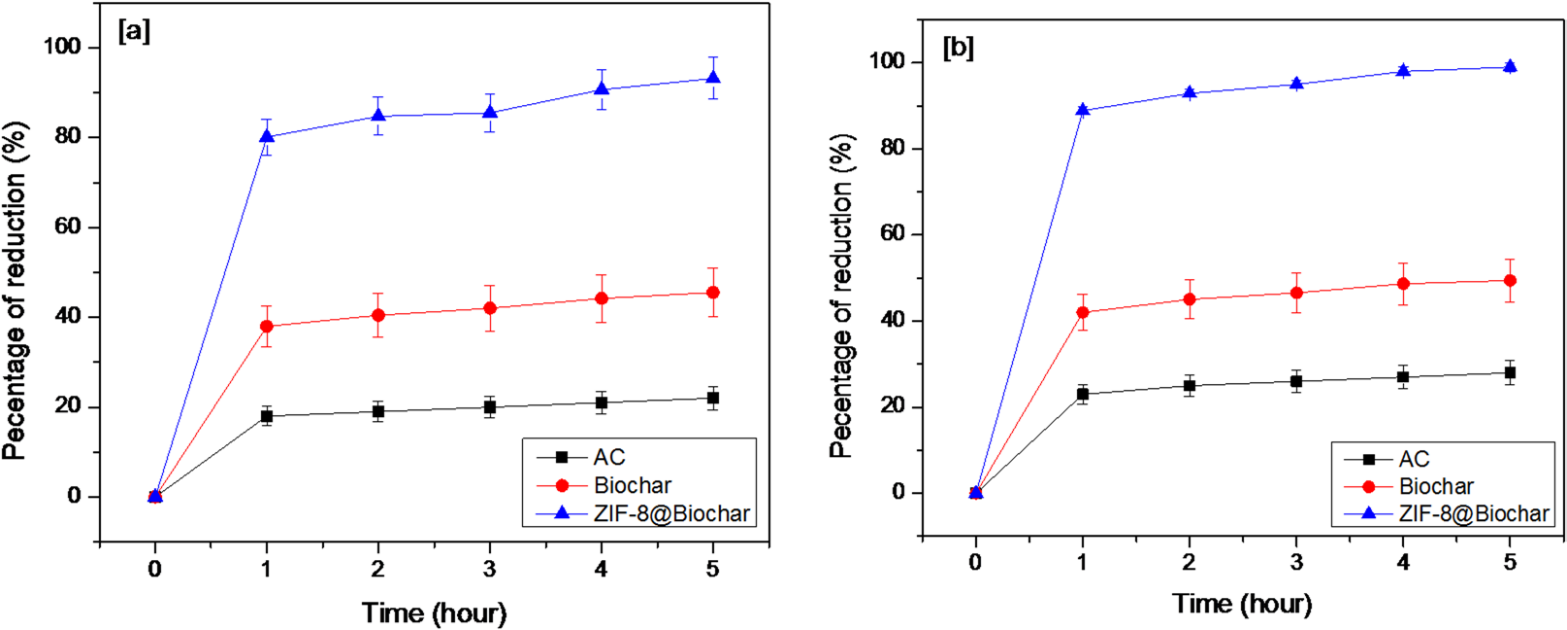
The significant reduction of acrylamide in [a] sunflower, [b] mixed oil.

After frying process for foods at high temperature, acrylamide was formed in oils due to Millard reactions between amino acids and sugars in the food being cooked^43^. The effect of activated carbon (AC), biochar, and ZIF-8@Biochar on acrylamide removal on different times in sunflower and mixed ‎oils results were recorded in Fig. 5. The results showed that positive relationship between times and acrylamide concentration when activated carbon (AC), biochar, and ZIF-8@Biochar were used to remove acrylamide. Two types of oil were used (sunfollower and mixed oil from soyabean and sunfolower). The reduction of acrylamide concentrations were 22%, 44.5%, and 92.5% at 5 h of treatment with activated carbon (AC), biochar, and ZIF-8@Biochar, respectively, in the case of sunfollower oil (Fig. 5a). However, in the case of mixed oils, the reductions were 27%, 49.5% and 99.9% at 5 h of treatment with activated carbon (AC), biochar, and ZIF-8@Biochar, respectively in the case of mixed oil (Fig. 5b). Our result agree with Chathiran et al., (2024)^44^ who revealed that acrylamide decreased in fried potatoes oil in to half when used biochar. The major mechanisms for acrylamide reduction from oil by biochar were physical ‎process and surface functionality^45^. The internal surface area and number of residual pores, which keep oil by capillary action, were the direct causes of the acrylamide reduction^46^. The adsorption of acrylamide depend on functional groups in biochar, Ambaye et al. (2020)^47^ showed that functional groups contain oxygen in structure including COOH allow biochar to bond with different compounds.

### Determination of acid value

**Fig. 6:**
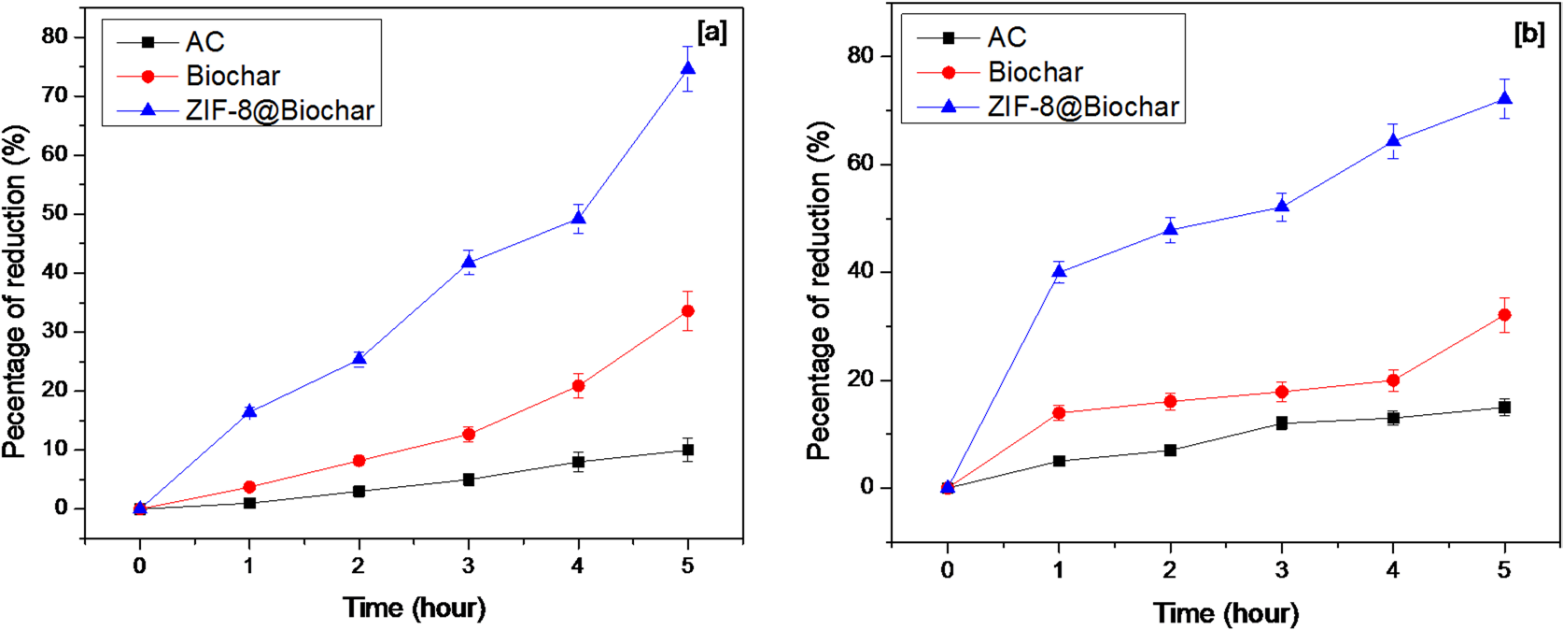
The significant reduction of acid values[a] sunflower, [b] mixed oil.

Free fatty acid (FFA) of the samples was measured as % reduction equivalents, and some significant differences were observed. Figure 6 shows the reduction of acid value in sunflower and mixed oils. The acid values on sunflower and mixed oils were depended on time when using AC, biochar, and ZIF-8@ biochar as adsorbent. The acid values results showed significant reduction at 9.7%, 33.9% and 74.8%, for AC, biochar, and ZIF-8@ biochar, respectively, in the case of sunflower oil. The acid values were 15.8%, 33.2% and 72.7% when AC, biochar, and ZIF-8@ biochar, respectively, were used in removal of acidity from mixed oils. The reduction of acid value in oil treated with AC, biochar and ZIF-8@ biochar‎ due to loss of free fatty acids for balance hydrolysis triglyceride and degradation reactions^48^.

### Determination of peroxide value

**Fig. 7:**
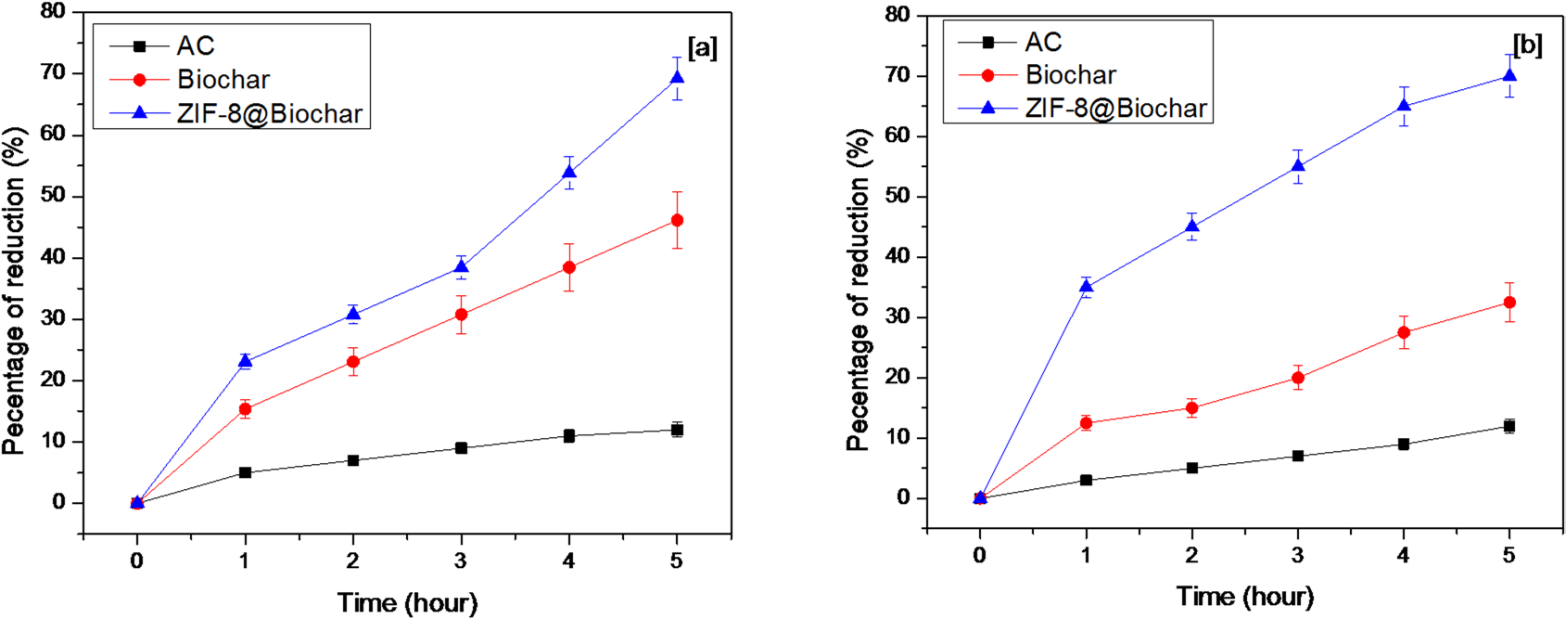
The reduction of peroxide values in[a] sunflower, [b] mixed oil.

Figure 7 shows the reduction of peroxide values in sunflower and mixed oils. As an important oil oxidation parameter, the peroxide values were also measured. The significant reduction of peroxide values refers to degradation of peroxides in sunflower, and mixed oil. Figure 7 showed that peroxides value on sunflower and mixed oils in time by using AC, biochar and ZIF-8@ biochar. The peroxides values were lowered in a time by AC, biochar and ZIF-8@ biochar. The reduction percentage of peroxides values in sunflower oil were 12.2%, 46.1%, and 69.7% at 5 h, when AC, biochar and ZIF-8@ biochar were used, respectively. The mixed oil results showed reductions at 12.4%, 32.6% and 70.4%, when AC, biochar and ZIF-8@ biochar were used at 5 h, respectively. Our results were agree with previous study in MOFs in which used in purify some vegetable oils from peroxides^33^. In that study, around 20–93% reductions in peroxide values with three different MOFs in sunflower, olive and linseed oils were reported^33^. The results also agreed with Chathiran et al., (2024)^44^. The significant reduction of peroxide values due to degradation of peroxides. The reduction of peroxide values increases physicochemical quality ‎of oils.

### Adsorption mechanism

The adsorption of acrylamide onto ZIF-8@biochar is primarily driven by a combination of electrostatic attraction, hydrogen bonding, and coordination interactions. ZIF-8’s porous structure and the presence of zinc ions (Zn^2+^) and imidazole linkers facilitate these interactions with acrylamide molecules. The positively charged zinc ions within the ZIF-8 structure can attract the negatively charged portions of the acrylamide molecule, leading to electrostatic interactions. The nitrogen and oxygen atoms present in the acrylamide molecule can form hydrogen bonds with the nitrogen atoms of the imidazole linkers in ZIF-8.The zinc ions in ZIF-8 can also act as Lewis acid sites and coordinate with the nitrogen or oxygen atoms of acrylamide, forming a coordinate bond. Acrylamide (AA) adsorption onto biochar primarily occurs through physical and chemical interactions involving the biochar’s pore structure and surface functional groups. The porous nature of biochar provides a large surface area for adsorption, while surface functional groups, like carboxyl, hydroxyl, and amino groups, can interact with acrylamide molecules. Biochar’s porous structure allows acrylamide molecules to be adsorbed into the pores, effectively increasing the surface area available for adsorption. Weak attractive forces between the biochar surface and acrylamide molecules can also contribute to adsorption. Functional groups like hydroxyl and amino groups on the biochar surface can form hydrogen bonds with acrylamide molecules.

**Fig. 8:**
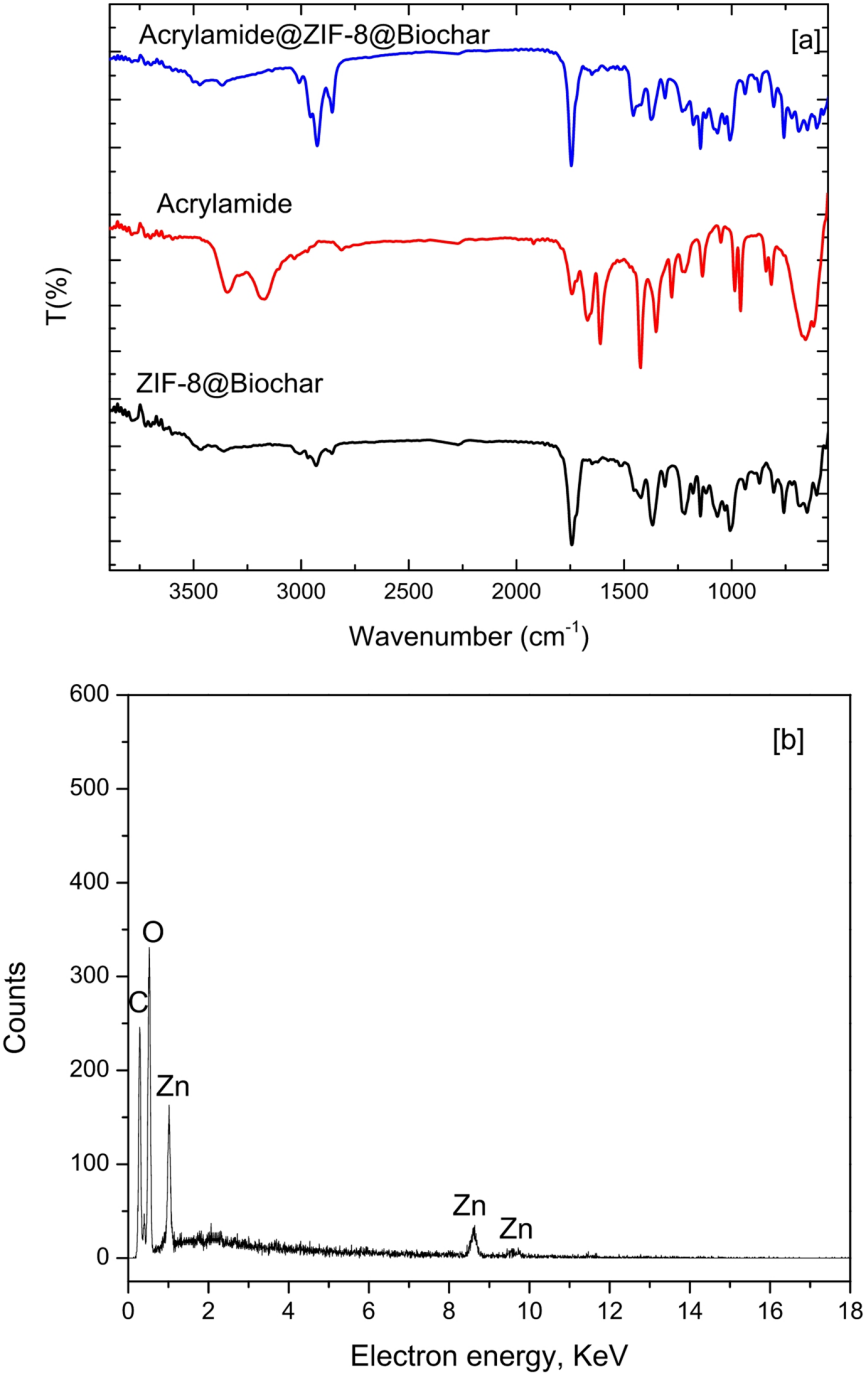
[a] FTIR of ZIF-8@Biochar before and after acrylamide adsorption from oils; [b] EDX of ZIF-8@Biochar before and after acrylamide adsorption from oils.

**Fig. 9:**
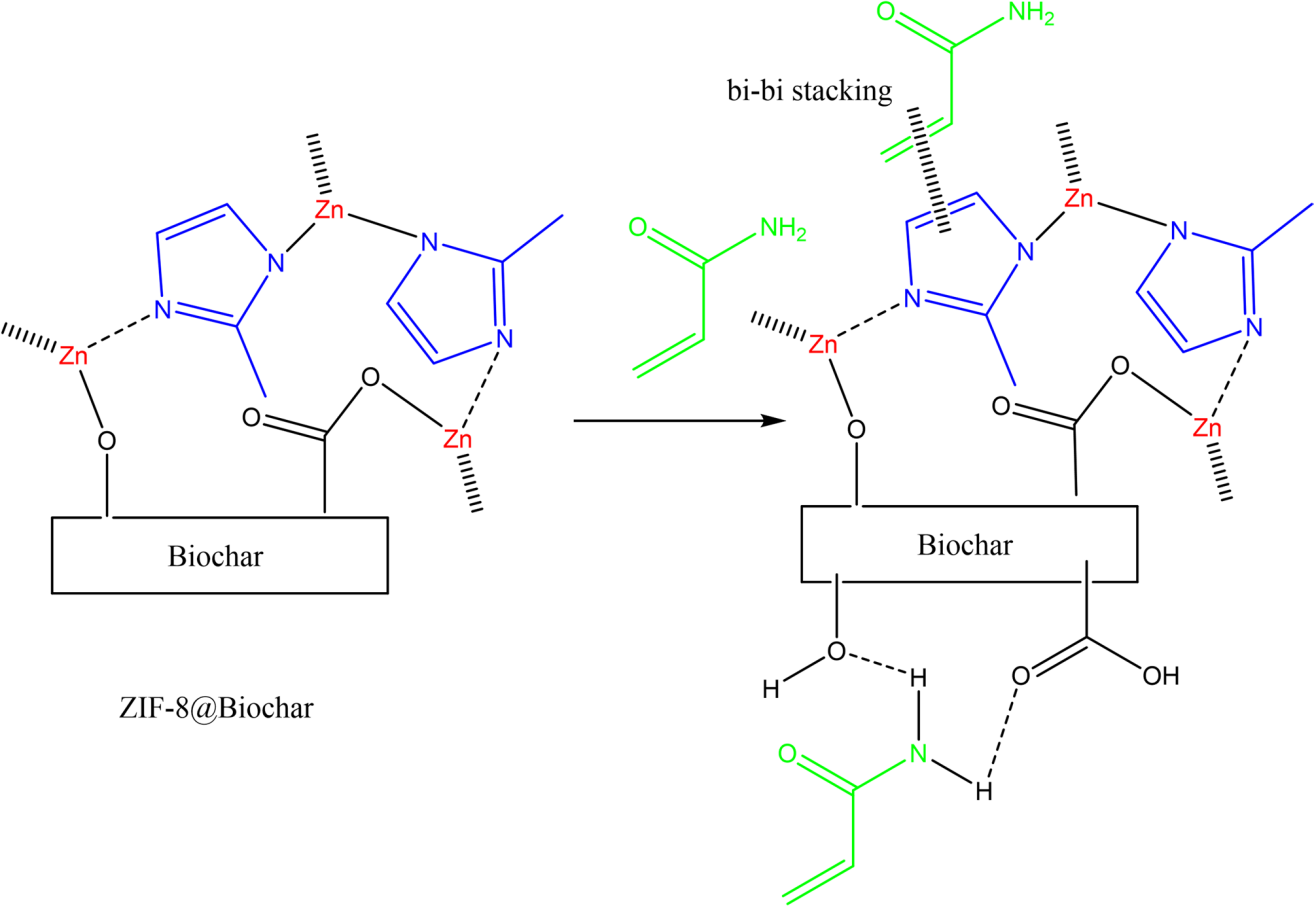
Proposed mechanism of acrylamide adsorption using ZIF-8@Biochar.

The mechanism of acrylamide adsorption using ZIF-8@Biochar was confirmed using FTIR spectra and EDX analysis. The disappearance of NH_2_ band which exhibits a characteristic peak between 3300 and 3500 cm^− 1^ confirms hydrogen bonding mechanism. The peak attributed to the N-H stretching vibration was shifted due to the interaction between acrylamide and ZIF-8@Biochar as shown in Fig. 8a. After the ZIF-8@Biochar composite has been used to adsorb acrylamide from oil, the EDX analysis showed distinct changes that help confirm that adsorption occurred and suggest the possible mechanism. EDX analysis of ZIF-8@Biochar composite after used to adsorb acrylamide from oil showed zinc (Zn), nitrogen (N), and additional carbon (C) and oxygen (O). EDX showed increased in nitrogen content due to adsorb acrylamide molecule and changes in carbon and oxygen content due to adsorb acrylamide (Fig. 8b). The possible interaction between ZIF-8/Biochar and acrylamide molecule was illustrated in Fig. 9.

## Conclusion

In this study, ZIF-8@Biochar was synthesized with in-situ technique and ZIF-8@Biochar was evaluated as adsorbent materials to remove acrylamide, acidity and peroxide values from used cooking oil. The activity of ZIF-8@Biochar was compared with active carbon as a conman adsorbent. The results showed that the composite ZIF-8@Biochar exhibited superior adsorption performance for acrylamide compared to both pure biochar and activated carbon due to enhance porosity, surface area, and stability. Acrylamide reduction rate of ZIF-8@Biochar reached 99.9%. Acidity and peroxide values increase as used cooking oil degrades, ZIF-8@Biochar showed 70% reduction in both acidity and peroxide values, indicated the excellent efficacy process. Overall, this study proves an important potential of ZIF-8@Biochar MOF to remove acrylamide, high acid and peroxide values from used cooking oil by adsorption principle. It pointed out that more research needed to design and synthesize new MOF structures for applying in the edible oil industry.

## Data Availability

The all data generated or analyzed during the current study are included in this manuscript.
